# Supplementary material for: The synthetic histone-binding regulator protein PcTF activates interferon genes in breast cancer cells
Source: BMC Syst Biol. 2018 Sep 25;12:83. doi: 10.1186/s12918-018-0608-4 (PMC6156859; doi:10.1186/s12918-018-0608-4)
Supplement: Supplementary file 1 — Figure S1. Comparisons of gene sets that are differentially or similarly expressed in BT-474, MCF7, BT-549, and MCF10A. Figure S2. Comparisons, by cell line, of expressed and silenced genes within PRC-modules. Figure S3. Jensen Shannon divergence analyses of transcription profiling data (RNA-seq) for all PcTF-treated and untreated cell samples. Figure S4. Detailed view of the transcription factor (TF) binding motif overrepresentation plot from Figure 3D. Figure S5. Expression levels of putative regulators of PUGs. Figure S6. Chromosome plot of PcTF-responsive genes that were identified in the RNA-seq experiment. Figure S7. Detailed view of MCF7 ChIP-seq signals. Table S1. The set of 45 H3K27me3-enriched, repressed (FPKM < 2) genes shared by the three cancer cell lines. Table S2. TF motif enrichment analysis results for the data shown in Fig. 3d. Table S3. Primers used to generate the RT-qPCR results shown in Fig. 6. (DOCX 3056 kb) [file 12918_2018_608_MOESM1_ESM.docx]

**SUPPORTING INFORMATION**

**Figure S1.** Comparisons of gene sets that are differentially or similarly expressed in BT-474, MCF7, BT-549, and MCF10A.

**Figure S2.** Comparisons, by cell line, of expressed and silenced genes within PRC-modules.

**Figure S3.** Jensen Shannon divergence analyses of transcription profiling data (RNA-seq) for all PcTF-treated and untreated cell samples.

**Figure S4.** Detailed view of the transcription factor (TF) binding motif overrepresentation plot from Figure 3D.

**Figure S5.** Expression levels of putative regulators of PUGs.

**Figure S6**. Chromosome plot of PcTF-responsive genes that were identified in the RNA-seq experiment.

**Figure S7.** Detailed view of MCF7 ChIP-seq signals.

**Table S1.** The set of 45 H3K27me3-enriched, repressed (FPKM < 2) genes shared by the three cancer cell lines.

**Table S2.** TF motif enrichment analysis results for the data shown in Figure 3D.

**Table S2.** Primers used to generate the RT-qPCR results shown in Figure 6.


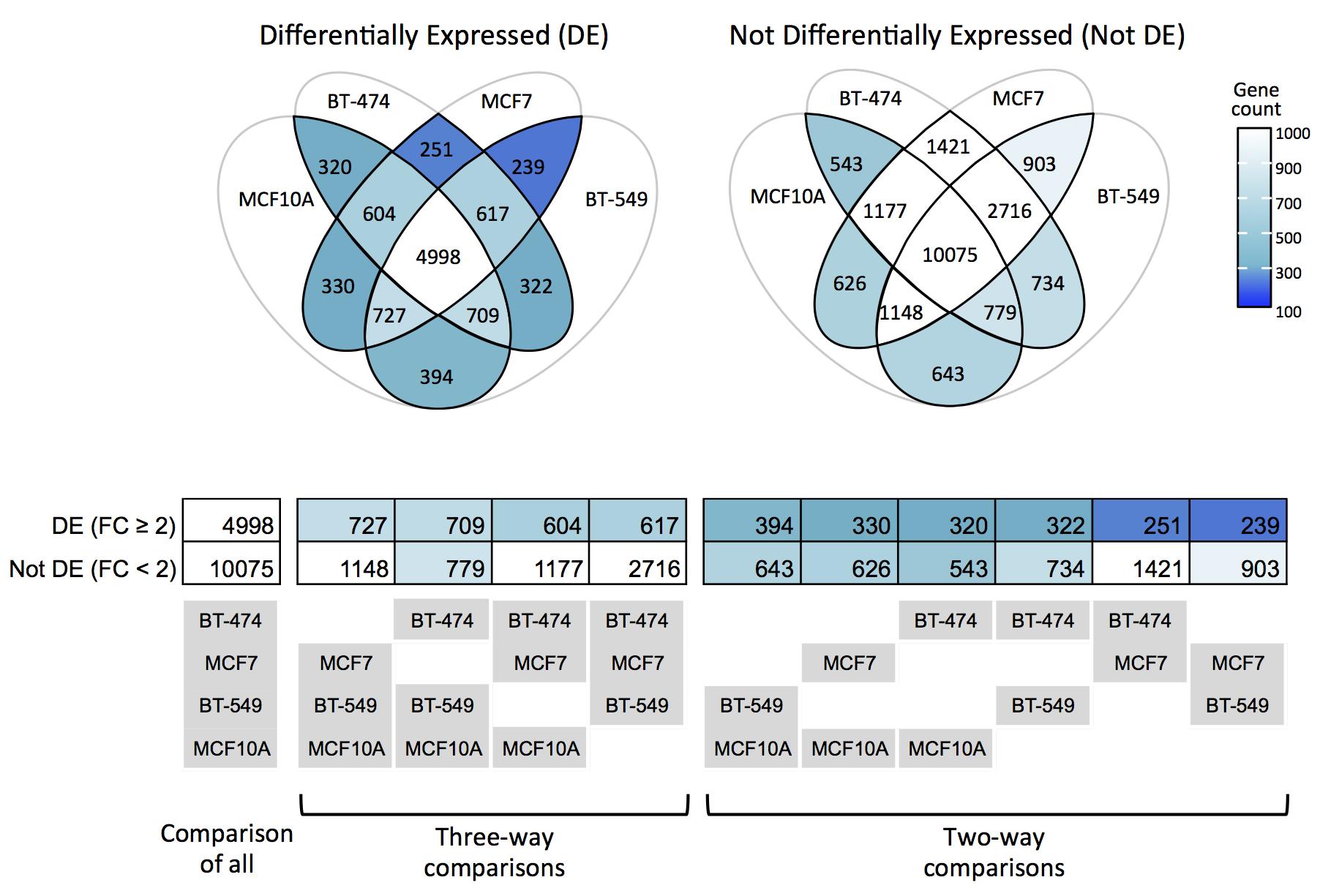


**Figure S1.** Comparisons of gene sets that are differentially or similarly expressed in BT-474, MCF7, BT-549, and MCF10A. Cuffdiff was used to determine genes with 2-fold or higher (*q* value ≤ 0.05) differential expression (DE) or genes with less than 2-fold differential expression (Not DE, expressed at similar levels). In the Venn diagrams, the numbers of DE or Not DE genes are shown in the pairwise overlap sectors, as well as in the table below.


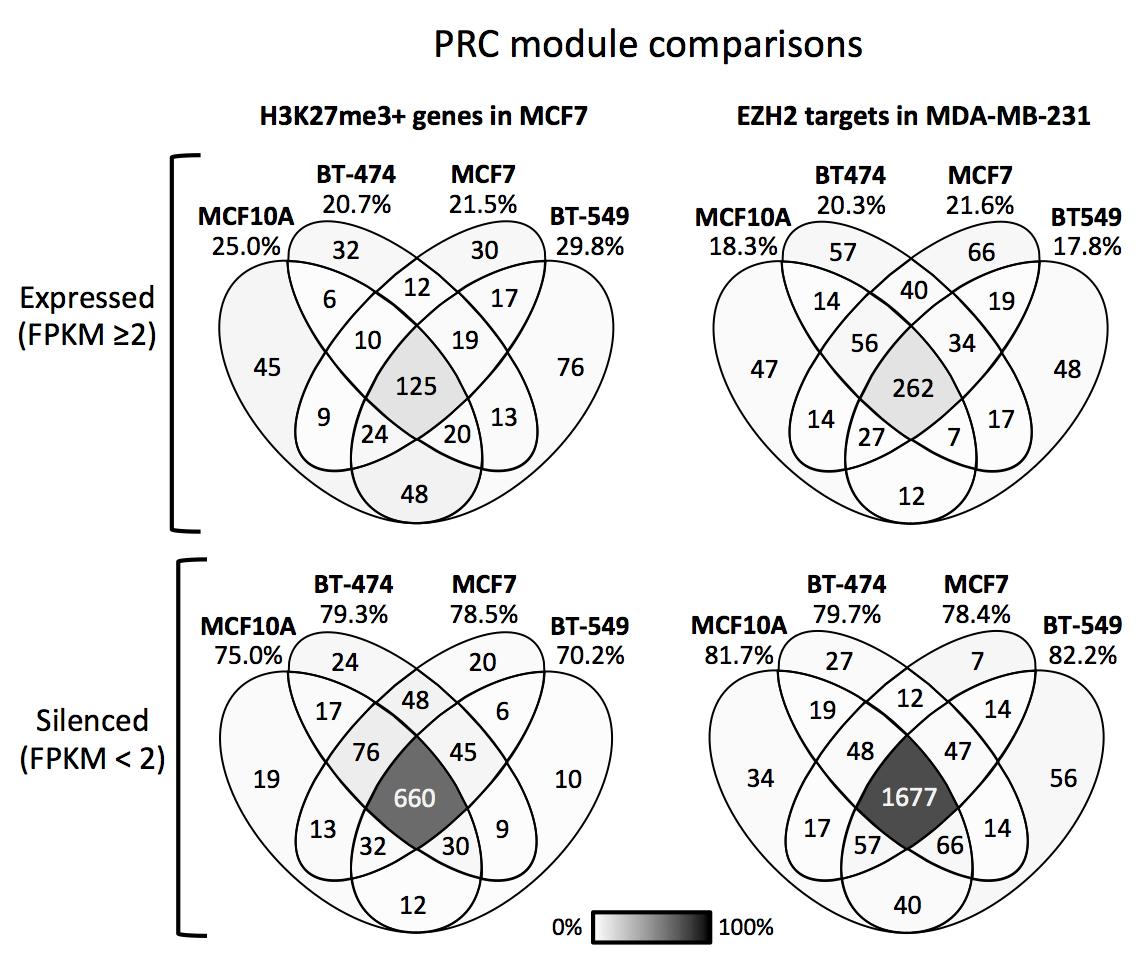


**Figure S2.** Comparisons, by cell line, of expressed and silenced genes within PRC-modules. The Venn diagram includes transcripts from Figure 2B (center and right-most box plots). Genes are categorized by expression level: expressed (FPKM ≥ 2) or silenced (FPKM < 2). The percentages of genes in each category (expressed or silenced) is shown under the label for each cell line.


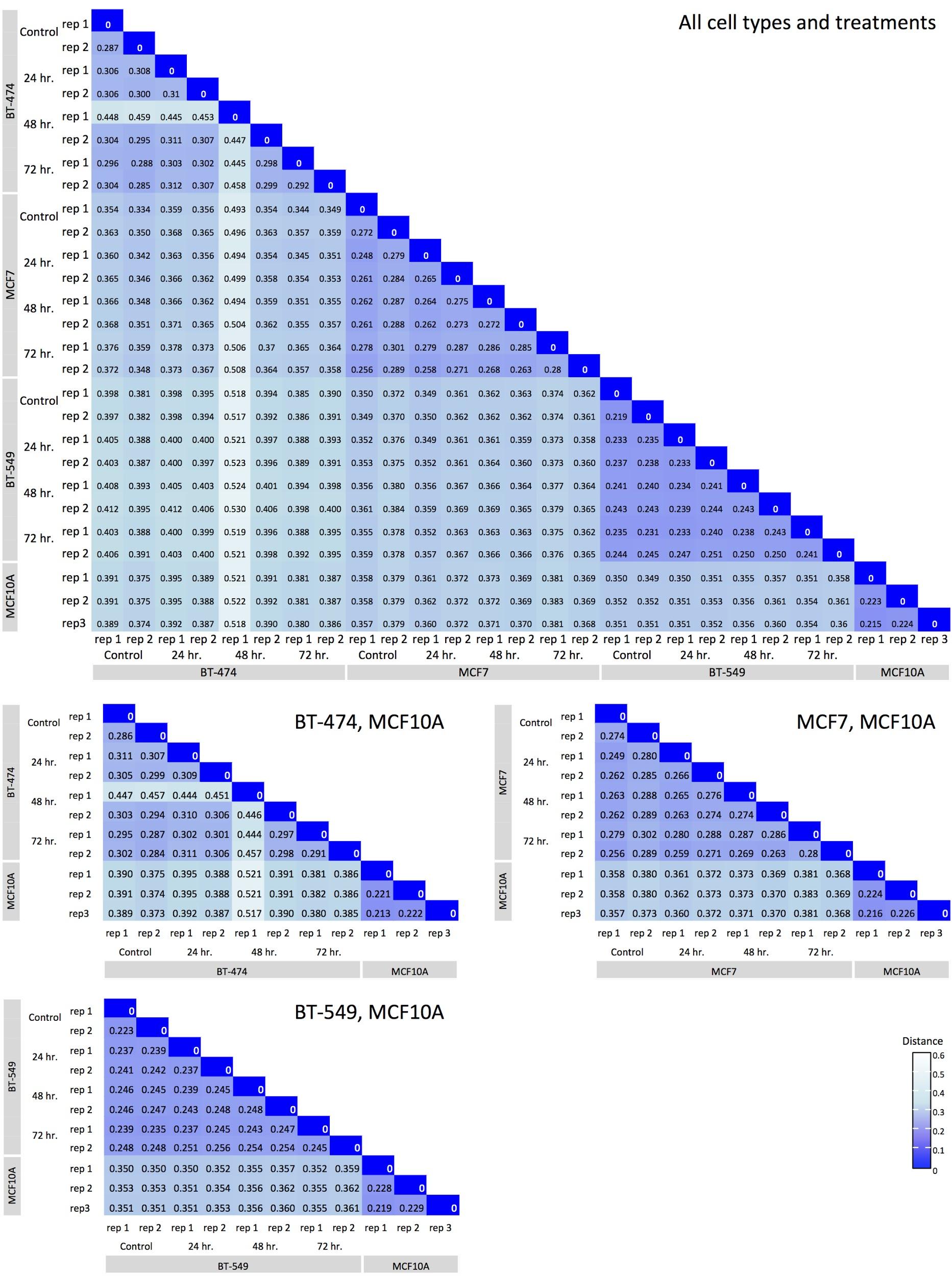


**Figure S3.** Jensen Shannon divergence analyses of transcription profiling data (RNA-seq) for all PcTF-treated and untreated cell samples. Jensen-Shannon Divergence (JSD) values were calculated as described for the data shown in Figure 2: similarity of the probability distributions of expression levels (FPKM values) for 63,286 total transcripts, which include 22,267 protein-coding transcripts.


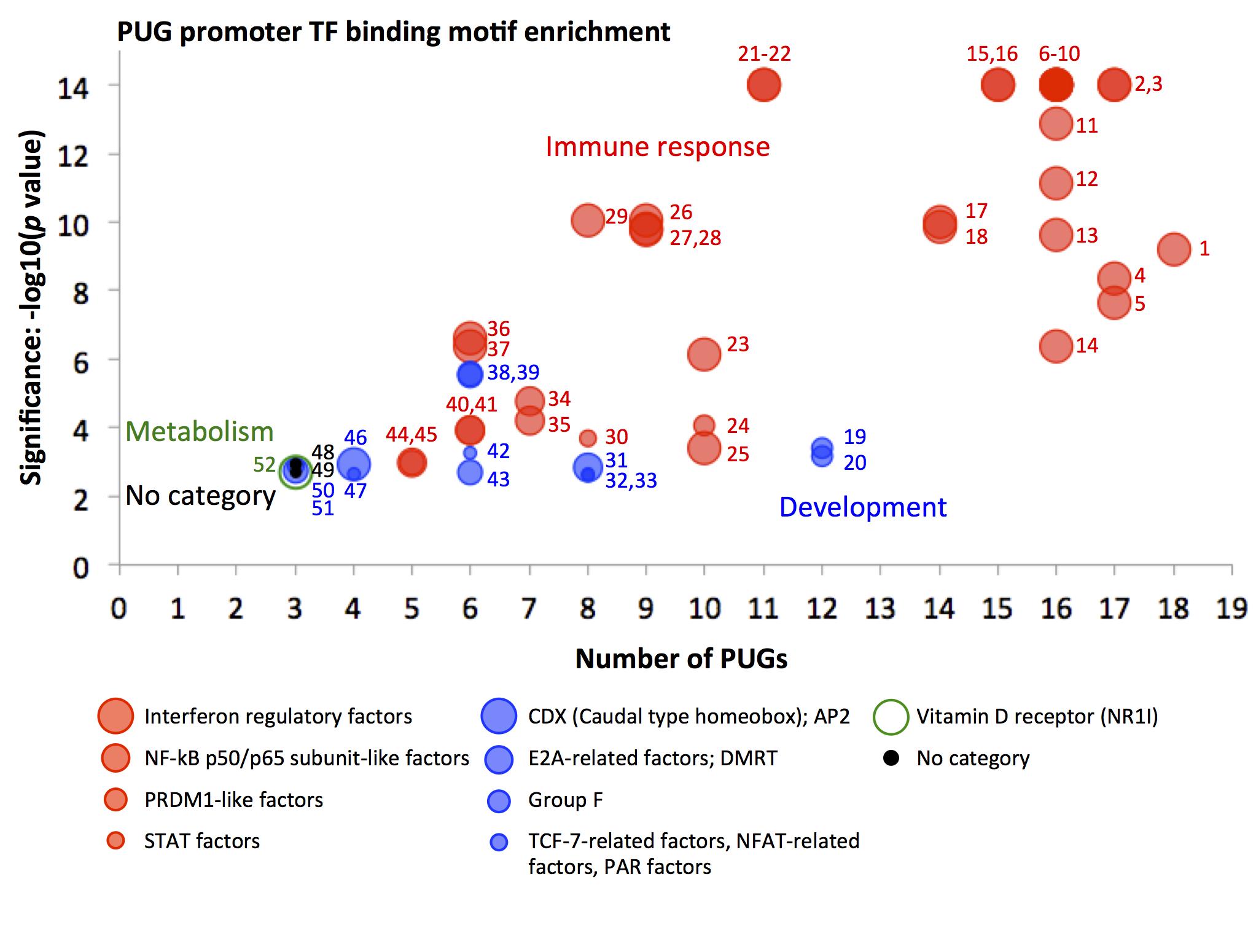


**Figure S4.** Detailed view of the transcription factor (TF) binding motif overrepresentation plot from Figure 3D. Each bubble (labeled with a numeral 1-52, right to left) represents a subset within the 19 common PcTF-upregulated genes (PUGs). The number of genes within each PUG subset is indicated on the x-axis. The genes within each subset have promoters that share a common transcription factor motif (see Table S2). Arbitrary bubble diameters vary by TF family (see legend). The y-axis values are negative, log10 transformed *p* values of binding motif overrepresentation in each PUG subset. Each *p* value is the hypergeometric cumulative distribution function (CDF) for enrichment of the PUG set with known TF target genes.

**
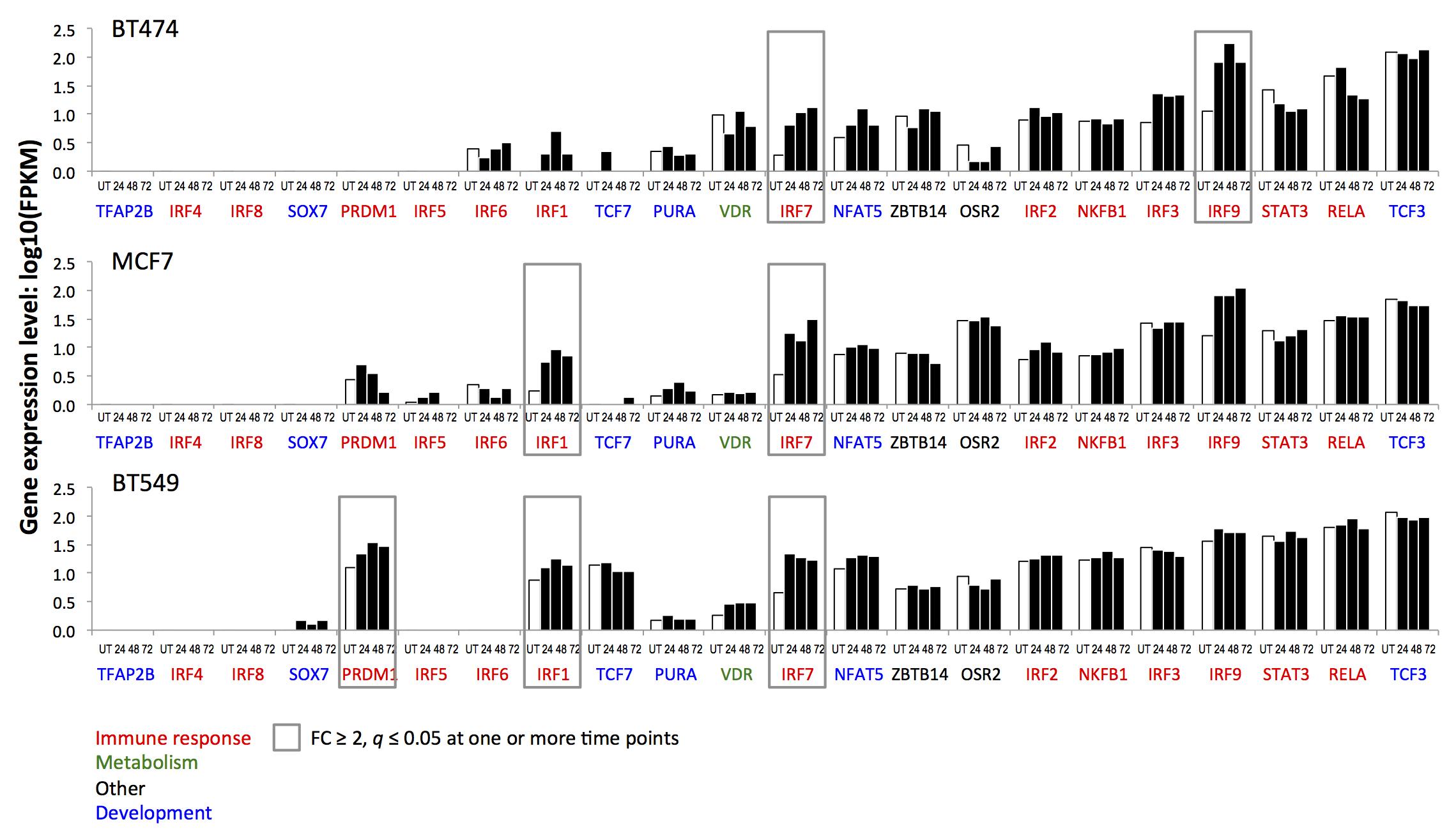
**

**Figure S5.** Expression levels of putative regulators of PUGs. Grey boxes indicate genes that become significantly upregulated (FC ≥ 2, *q* ≤ 0.05) at one of more time points in any cell type after PcTF expression. UT, untreated (white bars); 24, 48, and 72 indicate treatment timepoints in hours (black bars, left to right).


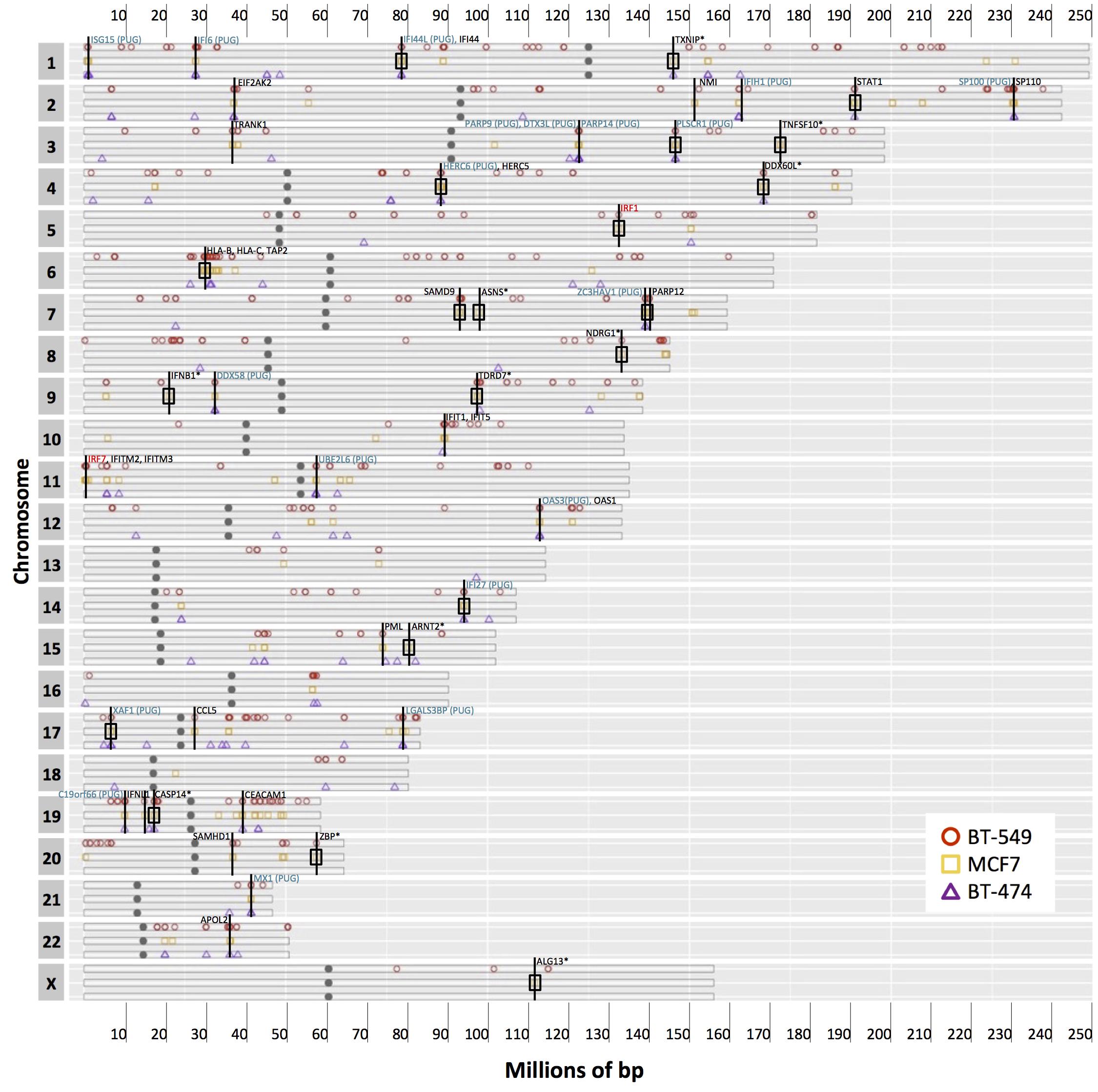


**Figure S6**. Chromosome plot of PcTF-responsive genes that were identified in the RNA-seq experiment. Colored, open shapes mark the positions of the transcription start sites of genes that show at least 2-fold upregulation (*q* ≤ 0.05) at one or more time points in BT-549, MCF7, or BT-474 cells. Dark filled circles represent the positions of centromeres. Coordinates are based on the Human Dec. 2013 (GRCh38/hg38) Assembly. Gene symbols (*ISG15*, *IFI6*, etc.) are shown for genes that are upregulated in MCF7: turquoise, PUGs; black, upregulated at all three time points in MCF7 only; asterisk (*), upregulated at one or two time points in MCF7; red, two putative regulators of PUGs, IRF1 and IRF7. Boxes mark the regions identified as having the highest (top 20%) mean enrichments for H3K27me3 (Fig. 4, Fig. S6) in MCF7.


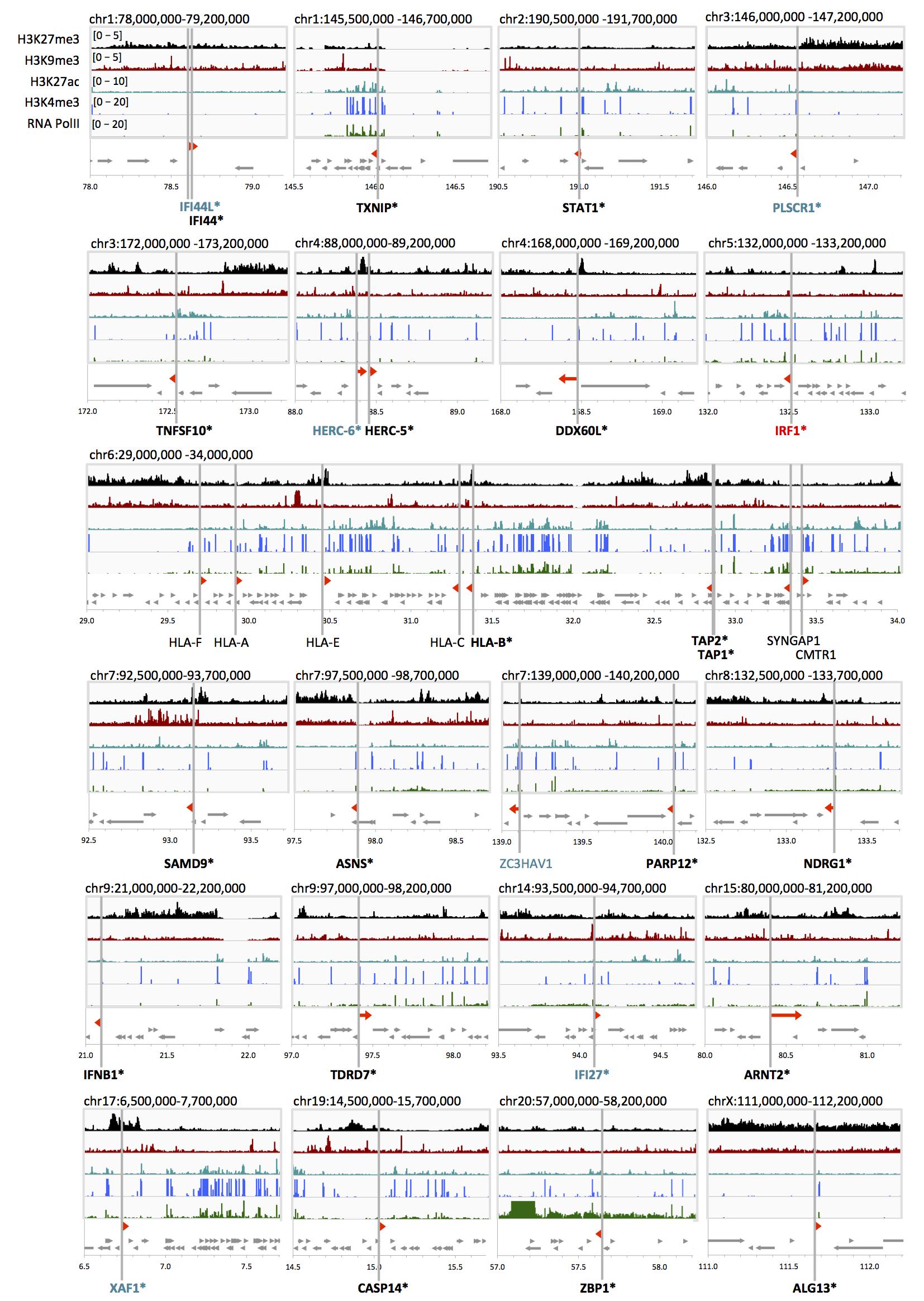


**Figure S7.** Detailed view of MCF7 ChIP-seq signals. Regions include PcTF-upregulated genes at loci with the highest (top 20%) mean enrichments for H3K27me3 (Fig. 4) in MCF7 cells. Bar charts show signal intensity (y-axis, range shown in brackets in the first chart) with a sliding window of 50 bp. Source files are the same as those used to generate the TSS plots in Figure 4.

|  | **PcTF: FC ≥ 2, q ≤ 0.05** | | |
| --- | --- | --- | --- |
| **Genes** | **BT-474** | **MCF7** | **BT-549** |
| AASS, ABCA6, ADRB2, ALOXE3, BACH2, BCL11A, BNC1, BTC, CCND2, CCNO, CDH13, CLYBL, CPVL, DCN, EGFLAM, ETV4, GJA3, GJB2, GRHL3, HES7, HOXA1, ITGA1, KLHL29, LIPG, LRRK2, LYSMD2, MCIDAS, PDE8B, PID1, PLXNA2, PPP1R14C, PRICKLE1, PRSS12, PTCHD4, SGPP1, SLC44A5, SNCA, SOX7, SPRR2D, SVEP1, THSD1, TMOD2, UNC5B, XKR6, ZBTB16 | none | none | PID1 (48, 72 hrs) |

**Table S1.** The set of 45 H3K27me3-enriched, repressed (FPKM < 2) genes shared by the three cancer cell lines (Fig. 1). Time points that showed significant upregulation (FC ≥ 2, *q* ≤ 0.05) for each gene in PcTF-expressing cells are shown.


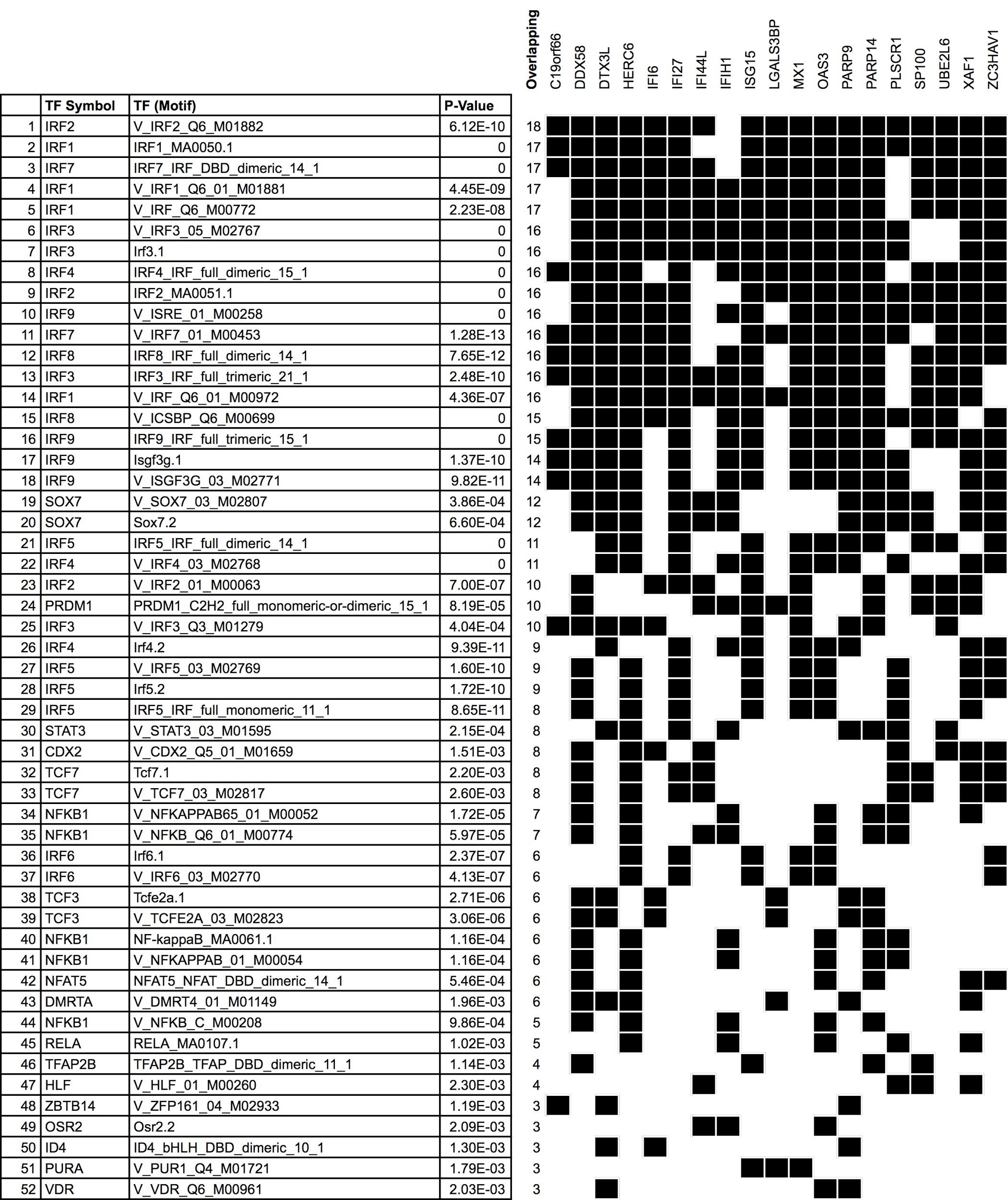


**Table S2.** TF motif enrichment analysis results for the data shown in Figure 3D. Column 1 values correspond to the data point labels in the plot shown in Figure S4. The symbols in the Motif column can be used to retrieve each sequence logo at <http://tfbsdb.systemsbiology.net/> using the “Search by TF” option. Column 2: TF Symbol - Gene symbol for the putative transcription factor regulator of the genes in input list (19 PUGs); Column 3: TF (Motif) - Motif name (nomenclature from motif repositories JASPAR, SELEX, TRANSFAC, and UniPROBE); Column 4: P-Value - Hypergeometric cumulative distribution function (CDF) for enrichment of the PUG list with TF target genes; Column 5: Overlapping - Gene symbols of PUGs from the input list (19 total) that overlap with all predicted targets of the TF (columns 2, 3).

| **Target gene** | **mRNA ID** | **Forward (5’..)** | **Reverse (5’..)** | **UPL Probe** |
| --- | --- | --- | --- | --- |
| mCherry (PcTF, Pc_Δ_TF) | n/a | CCTGAAGGGCGAGATCAAG | TTGACCTCAGCGTCGTAGTG | 41 |
| XAF1 | NM_017523.4 | AAGATCTCCTCCCTCCCTGA | GCTTTCGGTTGAGTTTCGTT | 23 |
| SAMD9L | NM_152703.4 | TTGTACCCCTTGCCCTTCT | GAATGGAGAGAGTTCAAGTTTATGATT | 1 |
| GBP1 | NM_002053.2 | ACTCAGGAAATCTCATAAGCTGGT | CTGCTGGTCATCTGGAAGAAT | 2 |
| CEACAM1 | NM_001712.4 | GATTTGCCATAGCCTTGAGGT | GGCATTACTGCCTTTACTTTCTCT | 72 |
| CASP14 | NM_001775.3 | AGTAGAAAGACCAGGAGGAGCTT | GAGCTGCCTCTAAATTTCTGTGA | 11 |
| SP100 | NM_001080391.1 | GAGGATGGGAAGAGGGAGAG | TCTTGTCACGGGTTTGTTGT | 9 |

**Table S3.** Primers used to generate the RT-qPCR results shown in Figure 6. UPL probe = Roche universal probe library LNA oligomer. The Roche UPL assay design tool and the mRNA ID’s shown here were used to generate primer sequences.
